# Serum Immunoglobulin M Concentration Varies with Triglyceride Levels in an Adult Population: Tianjin Chronic Low-Grade Systemic Inflammation and Health (TCLSIHealth) Cohort Study

**DOI:** 10.1371/journal.pone.0124255

**Published:** 2015-04-27

**Authors:** Hongbin Shi, Xiaoyan Guo, Qing Zhang, Hongmei Wu, Huanmin Du, Li Liu, Chongjin Wang, Yang Xia, Xing Liu, Chunlei Li, Shaomei Sun, Xing Wang, Ming Zhou, Qiyu Jia, Honglin Zhao, Kun Song, Dianjun Wei, Kaijun Niu

**Affiliations:** 1 The Second Hospital of Tianjin Medical University, Tianjin, China; 2 Health Management Centre, Tianjin Medical University General Hospital, Tianjin, China; 3 Nutritional Epidemiology Institute and School of Public Health, Tianjin Medical University, Tianjin, China; Baylor College of Medicine, UNITED STATES

## Abstract

Persistent low-grade inflammation is thought to underlie the pathogenesis of many chronic diseases, such as cardiovascular diseases and metabolic syndrome. Autoimmunity is correlated with increased levels of chronic low-grade inflammation, and immunoglobulin M (IgM) is reactive to autoantigens and believed to be important for autoimmunity. Triglyceride (TG) is fatty acid carrier and initiator of oxidative stress, and it has been hypothesized that TG stimulates B cells to secrete IgM. However, few studies have investigated the relationship between TG and IgM in human populations. We designed a cross-sectional and prospective cohort study to evaluate how serum TG levels are related to IgM concentration. Participants were recruited from Tianjin Medical University General Hospital-Health Management Centre. Both a baseline cross-sectional (n = 10,808) and a prospective assessment (n = 2,615) were performed. Analysis of covariance was used in the cross-sectional analysis. After multiple adjustments for confounding factors, serum IgM level in the highest quartile of TG in males was significantly higher than levels in lower quartiles (*P* <0.05). There was no significant difference between the four quartiles in females (*P* = 0.91). In follow-up analysis, a multiple linear regression model showed a significant and positive correlation between changes in IgM levels and changes of TG concentration in males (*P* = 0.04, standard β coefficient = 0.882). This cross-sectional and cohort study is the first to show that serum concentration of IgM varies with TG levels in adult male populations. Further research is needed to explore the mechanism by which TG leads to increased IgM concentration.

## Introduction

Obesity, which is increasing globally at an alarming rate, is a major risk factor for chronic diseases, including metabolic syndrome (MS), type 2 diabetes (T2D), cardiovascular disease (CVD), and certain forms of cancer [1–3]. Chronic low-grade inflammation due to obesity is thought to underlie the pathogenesis of many chronic diseases [4–6].

Pancreatic beta cell insufficiency and insulin resistance can induce disorders of lipid metabolism and glycometabolism that are associated with chronic diseases such as T2D, as well as CVD, and development of these metabolic disorders is correlated with obesity [7, 8]. Cells of the innate and adaptive immune system infiltrate and incite inflammatory responses in insulin responsive tissues, such as visceral adipose tissue (VAT), which is present in greater quantities in obese individuals[9]. Similarly, in T2D, pancreatic islets show evidence of inflammation, such as increased levels of cytokine or chemokine expression, as well as immune cell infiltration [10]. However, the molecular pathways underlying the associations between inflammation and metabolic disorders are largely unknown.

Recent studies have suggested that the increase of IgM antibodies was associated with antigen exposure caused by obesity [11]. IgM is the first class of antibodies produced during a primary antibody response, and is predominantly produced by B-1 cells [12]. In the absence of stimulation by specific antigens, IgM is polyreactive not only to foreign antigens but also to phylogenetically conserved autoantigens, including nucleic acids, heat shock proteins, carbohydrates, and phospholipids. Moreover, since IgM has a relatively low affinity for modified self-components [9, 13, 14], it is believed to be important for progression of autoimmunity.

Triglyceride (TG) is fatty acid carriers that can release fatty acids into tissues via the action of lipoprotein lipase. The increased levels of TG may therefore lead to elevated fatty acid levels in tissues and blood. Some studies indicate that fatty acid-induced metabolic dysfunction or impaired insulin secretion is associated with obesity [15, 16]; for example, one animal study demonstrated that saturated fatty acids in VAT increased IgM levels via stimulation of the B cell Toll-like receptor 4 (TLR-4) in a manner similar to lipopolysaccharide [17]. This finding supports the hypothesis that increased TG levels would lead to increased IgM concentration. Moreover, IgM antibodies dominate the humoral response to oxidation specific epitopes [18], which are generated through oxidative stress. Increased serum TG levels are known to promote oxidative stress [19], which might lead to increased IgM concentration; however, few prospective studies have comprehensively evaluated the relationship between TG levels and IgM concentration in an adult population.

Here, we have designed a cross-sectional and cohort study to investigate whether increased TG serum levels can lead to elevated IgM concentrations.

## Methods

### Participants

Tianjin chronic low-grade systemic inflammation and health (TCLSIH or TCLSIHealth) cohort study is a large prospective dynamic cohort study focusing on the relationships between chronic low-grade systemic inflammation and the health status of a population living in Tianjin, China [20, 21]. Tianjin is a city of approximately 10.43 million inhabitants, located in the northeast of the North China Plain, facing the Bohai Sea [22]. Participants received invitations to participate (based on a random procedure) while taking routine health examinations (rather than for a specific health reason) that took place once per year at Tianjin Medical University General Hospital-Health Management Center, the largest and most comprehensive local physical examination center. Nearly all occupations are covered in this study, and we also included retired individuals living in residential communities. We therefore consider that study cohort used here is representative of the adult population receiving physical examination in Tianjin. The protocol of this study was approved by the Institutional Review Board of the Tianjin Medical University and participants gave written informed consent prior to participation in the study.

**Fig 1:**
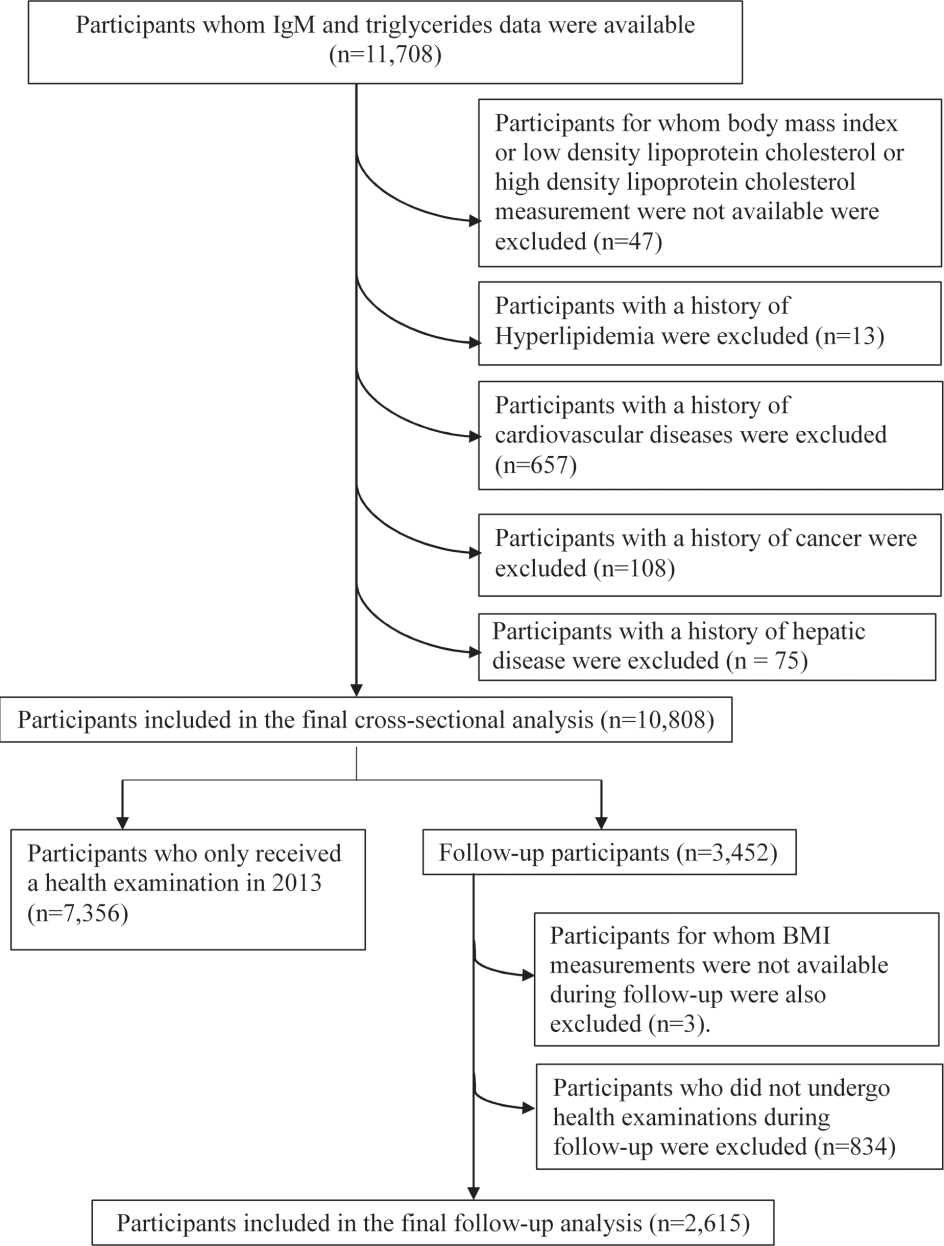
The participant selection process. Selection of the study population, Tianjin Chronic Low-grade Systemic Inflammation and Health (TCLSIHealth) Cohort Study, 2010 to 2013.

The TCLSIHealth data from 2010 to 2013 was used in this study. The participant selection process is described in **Fig 1**. During the research period there were 11,708 participants who had received at least one health examination including serum-immunological tests agreed to participate, and provided informed consent for their data to be analyzed. Participants whom body mass index (BMI) or high density lipoprotein cholesterol (HDL) or low density lipoprotein cholesterol (LDL) measurement were not available were excluded (n = 47). We excluded those with a history of Hyperlipidemia (n = 13) or CVD (n = 657) or cancer (n = 108) or hepatic disease (n = 75). Owing to these exclusions, the final cross-sectional study population comprised 10,808 participants (median (interquartile range, IQR) age: 46.0 (39.0, 54.0) years; male: 60.0%).

For follow-up analysis, participants were excluded at baseline if they had received a health examination only in 2013 (n = 7,356). The participants for whom BMI measurements were not available during follow-up were also excluded (n = 3). Following the exclusion, the final cohort study population comprised 2,615 participants (follow-up rate: 75.8%; mean (IQR) age: 45.0 (39.0, 52.0) years; male: 65.4%; followed up for 1~3 y, mean duration of follow-up (95% CI): 2.13 (2.10–2.16y)).

### Serum-immunological tests

Serum levels of IgM were determined by the immunonephelometric technique using the automated IMMAGE 800 immunochemistry system (Beckman Coulter, Brea, CA, USA), and expressed as mg/dL. The detection limit of the assay was 4.2 mg/dL; the measurement range was: 4.2–14,400 mg/dL; and the intra- and inter-assay coefficients of variation (CV) were less than 6% for IgM. The manufacturer indicates the following reference intervals for healthy adults: IgM 46–304 mg/dL.

### Assessment of other variables

Waist circumference was measured at the umbilical level with participants standing and breathing normally. Blood pressure (BP) was measured twice from the upper left arm using an automatic device (Andon, Tianjin, China) after 5 minutes of rest in a seated position. The mean of these 2 measurements was taken as the BP value. Blood samples for the analysis of fasting blood sugar (FBS) and lipids were collected in siliconized vacuum plastic tubes. FBS was measured by the glucose oxidase method, triglyceride (TG) was measured by enzymatic methods, low density lipoprotein cholesterol (LDL) was measured by the polyvinyl sulfuric acid precipitation method, and high-density lipoprotein cholesterol (HDL) was measured by the chemical precipitation method using reagents from Roche Diagnostics on an automatic biochemistry analyzer (Roche Cobas 8000 modular analyzer, Mannheim, Germany). Serum creatinine (SCr) was determined by the alkaline picrate Jaffé kinetic method with the Cobas 8000. Albumin (ALB) in serum was measured by the bromocresol green method with the Cobas 8000.

Anthropometric parameters (height and body weight) were recorded using a standard protocol. Body mass index (BMI) was calculated as weight/height^2^ (kg/m^2^). Participants were considered to have diabetes when their FBS accorded with level of ≥7 mmol/L or having a history of diabetes. Hypertension is defined as having a BP higher than 140/90mmHg (systolic blood pressure (SBP)/diastolic blood pressure (DBP)) or having a history of hypertension. Sociodemographic variables, including gender and, age were also assessed. A detailed personal and family history of physical illness and current medications were noted from ‘‘yes” or ‘‘no” responses to relevant questions. Information on alcohol and tobacco use were obtained from a questionnaire survey.

### Statistical Analysis

All statistical analyses were performed using the Statistical Analysis System version 9.3 for Windows (SAS Institute Inc., Cary, NC, USA). Because their IgM serum concentration was significantly different (see the results section), males and females were analyzed separately in this study. Descriptive data is presented as the median (IQR) for continuous variables with non-normal distributions and as percentages for categorical variables. Because the distribution of all continuous variables was non-normal, the natural logarithm was applied to normalize the data before statistical analysis. For baseline characteristics analysis, the differences among TG categories were examined using analysis of variance for continuous variables and multiple logistic regression analysis for proportional variables. Bonferroni-corrected P values were used for comparisons between TG quartiles. Analysis of covariance was used to examine relationships between quartiles of TG, and the level of IgM after adjustment for covariates: age, baseline BMI, SCr, ALB, hypertension, diabetes, smoking status, drinking status, and family history of CVD, hypertension, hyperlipidemia, and diabetes. A linear trend across increasing quartiles was tested by using the median value of each quartile as an ordinal variable. A Pearson’s correlation coefficient (r) was calculated to evaluate the relationship between two continuous variables. The multiple linear regression analysis was used to examine the relationships between the change of TG and the change of IgM with adjustment for the covariates mentioned above. All tests were two tailed and *P* <0.05 was defined as statistically significant.

## Results

60.0% of participants in this study were males and 40.0% females, with median (IQR) ages of 46.0 (39.0, 53.0) and 46.0 (39.0, 55.0) years, respectively. Characteristics of participants across TG quartiles for cross-sectional analysis are presented in **Table 1**. Males with a higher level of TG were younger (*P* for trend <0.0001). Compared with participants in the lowest TG quartile, participants in the upper three quartiles had higher BMI, waist circumferences, TC, SBP, DBP, FBS, SCr, and ALB, lower HDL, and a higher proportion of current smokers and drinkers. In the highest TG quartile, the level of IgM was significantly higher than the lower three quartiles (*P* <0.05). The proportion of subjects with diabetes, hypertension, and subjects with a family history of diabetes and hypertension were all significantly larger in the highest TG quartile (*P* for all trends <0.001). No significant differences in proportion of ex-smokers and family history of hyperlipidemia and CVD were observed across quartiles of TG. In females, compared with participants in the lowest quartile of TG, participants in the upper three quartiles had higher age, BMI, waist circumferences, TC, SBP, DBP, LDL, FBS and SCr, lower HDL and IgM, a higher proportion of current smokers (*P* <0.01). Adjusted geometric means (95% CI) of ALB levels across TG quartiles in males were 45.6 (45.4, 45.8) (g/L), 45.5 (45.3, 45.7) (g/L), 45.6 (45.4, 45.8) (g/L), 46.1 (45.9, 46.2) (g/L) (*P* for trend = 0.77), while in the highest TG quartile, the level of ALB was significantly higher than the lower three quartiles (*P* <0.05). The percentage of subjects with a family history of hypertension, and subjects who were drinkers were both significantly lower in the highest TG quartile (*P* for trend, 0.04 and 0.02, respectively). The proportion of subjects with diabetes and hypertension was significantly larger in the highest TG quartile (*P* for all trends <0.0001). Otherwise, no significant difference was observed between different quartiles of TG.

**Table 1:** Participant characteristics by quartiles of serum triglycerides concentration (n = 10,808) ^a^.

	Quartiles of serum triglycerides concentration (range, mmol/L)				
Males	Level 1 (0.32–1.14)	Level 2 (1.15–1.63)	Level 3 (1.64–2.37)	Level 4 (2.38–28.35)	*P* for trend ^b^
	(n = 1,635)	(n = 1,621)	(n = 1,595)	(n = 1,619)	
**Age (y)**	45.3 (38.0, 55.0) ^c^	46.3 (40.0, 54.0) ^d^	46.0 (40.0, 53.0) ^d^	45.0 (39.0, 51.0)	0.0012
**BMI (kg/m** ^**2**^ **)**	24.5 (22.5, 26.8) ^d^	25.7 (23.8, 28) ^d^	26.4 (24.7, 28.5) ^d^	27.1 (25.3, 29.1)	< 0.0001
**Waist circumference (cm)**	86.0 (80.0, 92.0) ^d^	90.0 (84.0, 96.0) ^d^	92.0 (87.0, 97.0) ^d^	94.0 (89.0, 99.0)	< 0.0001
**TC (mmol/L)**	4.74 (4.21, 5.30) ^d^	5.10 (4.56, 5.67) ^d^	5.30 (4.78, 5.90) ^d^	5.54 (4.92, 6.21)	< 0.0001
**LDL (mmol/L)**	2.90 (2.45, 3.40) ^d^	3.18 (2.70, 3.69) ^d^	3.21 (2.70, 3.75) ^d^	3.12 (2.46, 3.70)	< 0.0001
**HDL (mmol/L)**	1.40 (1.21, 1.63) ^d^	1.27 (1.09, 1.46) ^d^	1.19 (1.03, 1.37) ^d^	1.06 (0.92, 1.24)	< 0.0001
**SBP (mmHg)**	120 (110, 135) ^d^	125 (115, 135) ^d^	125 (115, 135) ^d^	130 (120, 140)	< 0.0001
**DBP (mmHg)**	80 (70, 85) ^d^	80 (75, 90) ^d^	85 (75, 90) ^d^	85 (80, 95)	< 0.0001
**FBS (mmol/L)**	4.9 (4.5, 5.3) ^d^	5.1 (4.6, 5.6) ^d^	5.1 (4.7, 5.6) ^d^	5.3 (4.9, 5.9)	< 0.0001
**ALB (g/L)**	46 (44, 48) ^d^	47 (45, 49)	47 (45, 49)	47 (45, 49)	< 0.0001
**SCr (μmol/L)**	77 (70, 85) ^d^	78 (71, 85)	79 (72, 86)	78 (71, 85)	< 0.0001
**IgM (mg/dl)**	79.1 (58.4, 107.0) ^d^	81.9 (59.5, 111.0)	80.4 (59.0, 108.0) ^d^	83.7 (61.6, 113.0)	0.71
**Hypertension (%)**	27.7	34.7	38.1	45.3	< 0.0001
**Diabetes (%)**	7.2	10.0	10.7	14.9	< 0.0001
**Smoking status (%)**					
** Smoker**	40.6	49.3	49.5	55.6	< 0.0001
** Ex-smoker**	0.31	0.12	0.31	0.06	0.25
**Drinker (%)**	60.1	65.4	68.8	76.3	< 0.0001
**Family history of diseases (%)**					
** CVD**	29.4	33.6	32.5	33.2	0.07
** Hypertension**	43.6	46.8	47.6	50.2	< 0.001
**Hyperlipidemia**	0.31	0.25	0.25	0.43	0.44
** Diabetes**	16.2	19.4	20.6	21.9	< 0.001
Females	**Level 1 (0.32–0.78)**	**Level 2 (0.79–1.09)**	**Level 3 (1.10–1.58)**	**Level 4 (1.59–23.74)**	
	**(n = 1,102)**	**(n = 1,079)**	**(n = 1,071)**	**(n = 1,086)**	
**Age (y)**	40.0 (35.0, 46.0) ^d^	46.0 (39.0, 53.0) ^d^	49.0 (41.0, 57.0) ^d^	52.0 (44.3, 60.0)	<0.0001
**BMI (kg/m** ^**2**^ **)**	21.9 (20.3, 23.9) ^d^	23.3 (21.4, 25.4) ^d^	24.4 (22.4, 26.6) ^d^	25.8 (23.5, 28.2)	<0.0001
**Waist circumference (cm)**	73 (68, 79) ^d^	77 (72, 83) ^d^	80 (75, 87) ^d^	84 (79, 91)	<0.0001
**TC (mmol/L)**	4.66 (4.12, 5.21) ^d^	5.01 (4.45, 5.62) ^d^	5.25 (4.65, 5.97) ^d^	5.69 (5.11, 6.39)	<0.0001
**LDL (mmol/L)**	2.66 (2.22, 3.13) ^d^	2.98 (2.49, 3.51) ^d^	3.17 (2.67, 3.82) ^d^	3.39 (2.79, 4.04)	<0.0001
**HDL (mmol/L)**	1.71 (1.48, 1.96) ^d^	1.60 (1.39, 1.82) ^d^	1.45 (1.26, 1.68) ^d^	1.28 (1.09, 1.47)	<0.0001
**SBP (mmHg)**	110 (100, 120) ^d^	120 (110, 130) ^d^	125 (110, 135) ^d^	130 (115, 145)	<0.0001
**DBP (mmHg)**	70 (65, 80) ^d^	75 (65, 80) ^d^	75 (70, 85) ^d^	80 (70, 85)	<0.0001
**FBS (mmol/L)**	4.7 (4.4, 5.0) ^d^	4.8 (4.5, 5.2) ^d^	4.9 (4.6, 5.3) ^d^	5.1 (4.7, 5.6)	<0.0001
**ALB (g/L)**	46 (44, 48) ^d^	46 (44, 48) ^d^	46 (44, 48) ^d^	46 (44, 48)	0.77
**SCr (μmol/L)**	57 (52, 64) ^d^	58 (53, 64)	58 (53, 65)	59 (54, 65)	0.0016
**IgM (mg/dl)**	124.0 (89.7, 169.0) ^d^	115.0 (86.0, 157.0) ^d^	107.0 (79.5, 149.0)	105.5 (75.3, 147.0)	<0.0001
**Hypertension (%)**	10.3	19.6	26.6	40.2	<0.0001
**Diabetes (%)**	1.3	2.9	6.1	9.1	<0.0001
**Smoking status (%)**					
** Smoker**	3.2	5.0	4.8	6.9	<0.01
** Ex-smoker**					
**Drinker (%)**	16.0	16.0	14.7	12.8	0.02
**Family history of diseases (%)**					
** CVD**	29.4	39.5	33.2	34.9	0.1
** Hypertension**	47.9	51.3	45.9	45.5	0.04
**Hyperlipidemia**	0.5	0.3	0.7	0.1	0.32
** Diabetes**	18.6	19.4	20.4	19.4	0.68

^a^ BMI, body mass index; TC, total cholesterol; TG, triglycerides; LDL, low density lipoprotein cholesterol; HDL, high-density lipoprotein-cholesterol; SBP, systolic blood pressure; DBP, diastolic blood pressure; FBS, fasting blood sugar; ALB, albumin; SCr, serum creatinine; IgM, immunoglobulin M; CVD, cardiovascular disease.

^b^ Analysis of variance or logistic regression analysis.

^c^ Median (interquartile range) (all such values).

^d^ Significantly different from the forth quartile of triglycerides (Bonferroni correction): P < 0.05.

**Table 2**shows the crude and adjusted relationships between quartiles of TG and IgM in participants. In the final multivariate models, adjusted IgM levels (95% CI) across TG quartiles in males were 76.6 (64.9, 90.3) (mg/dL), 78.9 (66.9, 93.1) (mg/dL), 78.8 (66.9, 93.0) (mg/dL), 82.8 (70.2, 97.7) (mg/dL) (*P* for trend = 0.08), respectively. IgM level in the highest quartile was significantly different from levels in the lower quartiles (Bonferroni-corrected *P* <0.05). In females, after adjustment, IgM levels (95% CI) across TG quartiles were 106.3 (93.9, 120.3) (mg/dL), 105.8 (93.6, 119.6) (mg/dL), 104.7 (92.7, 118.2) (mg/dL), 105.4 (93.2, 119.2) (mg/dL) (*P* for trend = 0.48), respectively. There was no significant difference between the four categories (*P* = 0.92).

**Table 2:** Adjusted relationships of quartiles of serum triglycerides concentration to immunoglobulin M (n = 10,808) ^a^.

	Quartiles of serum triglycerides concentration (range, mmol/L)					
Males	Level 1 (0.32–1.14)	Level 2 (1.15–1.63)	Level 3 (1.64–2.37)	Level 4 (2.38–28.35)	*P* value ^b^	*P* for trend ^b^
	(n = 1,635)	(n = 1,621)	(n = 1,595)	(n = 1,619)		
**IgM (mg/dl)**						
** Crude**	80.2 (78.4, 82.0) ^c^	80.7 (78.9, 82.6)	79.7 (77.9, 81.5) ^e^	83.7 (81.8, 85.6)	0.016	0.71
** Age-, and BMI-adjusted**	78.9 (77.1, 80.8) ^e^	80.8 (79.0, 82.7)	80.3 (78.5, 82.2)	84.3 (82.4, 86.3)	0.001	0.31
** Multiple adjusted** ^**d**^	76.8 (65.2, 90.6) ^e^	79.0 (67.0, 93.1) ^e^	78.3 (66.4, 92.3) ^e^	82.8 (70.2, 97.6)	<0.001	0.26
Females	**Level 1 (0.32–0.78)**	**Level 2 (0.79–1.09)**	**Level 3 (1.10–1.58)**	**Level 4 (1.59–23.74)**		
	**(n = 1,102)**	**(n = 1,079)**	**(n = 1,071)**	**(n = 1,086)**		
**IgM (mg/dl)**						
** Crude**	123.1 (119.6, 126.7) ^e^	114.3 (111.0, 117.7)	108.7 (105.6, 111.9)	105.1 (102.1, 108.2)	<0.0001	<0.0001
** Age-, and BMI-adjusted**	113.7 (110.3, 117.1)	112.6 (109.4, 115.8)	111.7 (108.6, 115.0)	112.6 (109.3, 116.0)	0.89	0.43
** Multiple adjusted** ^**d**^	107.0 (94.7, 121.0)	106.1 (93.9, 119.8)	105.3 (93.3, 118.8)	106.0 (93.9, 119.8)	0.91	0.46

^a^ BMI, body mass index; IgM, immunoglobulin M.

^b^ Analysis of covariance.

^c^ Adjusted geometric mean (95% confidence interval) (all such values).

^d^ Adjusted for age, BMI, albumin, serum creatinine, hypertension, diabetes, smoking status, drinking status, and family history of cardiovascular disease, hypertension, hyperlipidemia, and diabetes.

^e^ Significantly different from the forth quartile of triglycerides (Bonferroni correction): P < 0.05.

Baseline characteristics for follow-up analysis are shown in **Table 3**. In our cohort study, 65.4% of participants were males. The median (IQR) age of participants was 45.0 (39.0, 52.0) years (46.0 (39.0, 52.0) in males and 44.0 (38.0, 52.0) in females). Changes in IgM concentration were positive and significant correlated with changes in TG levels in males by Pearson’s correlation coefficient analysis (r = 0.045, *P* = 0.04), but not in females (r = 0.025, *P* = 0.46). After adjustment for age, linear regression model showed a positive and significant relationship between the two variables in males population (*P* = 0.04, standard β coefficient = 0.864). A multiple linear regression model showed a positive and significant relationship between changes in TG levels and changes in IgM concentration in males, after adjustment for potential confounding factors including age, baseline BMI, smoking status, drinking status, and family history of CVD, hypertension, hyperlipidemia, and diabetes (*P* = 0.04, standard β coefficient = 0.882) (**Table 4**).

**Table 3:** Cohort Analysis: Participant characteristics (n = 2615) ^a^.

	Males (n = 1710)	Females (n = 905)
**Age (y)**	46.0 (39.0, 52.0) ^b^	44.0 (38.0, 52.0)
**BMI (kg/m** ^**2**^ **)**	25.8 (23.8, 27.9)	23.6 (21.5, 25.8)
**Waist circumference (cm)**	90 (84, 96)	78 (71, 84)
**TC (mmol/L)**	5.15 (4.63, 5.79)	5.11 (4.52, 5.81)
**LDL (mmol/L)**	3.1 (2.59, 3.61)	3.03 (2.48, 3.59)
**HDL (mmol/L)**	1.26 (1.09, 1.47)	1.57 (1.34, 1.82)
**SBP (mmHg)**	125 (115, 135)	120 (105, 130)
**DBP (mmHg)**	80 (75, 90)	75 (65, 80)
**FBS (mmol/L)**	5.0 (4.5, 5.5)	4.8 (4.4, 5.1)
**IgM (mg/dl)**	76.0 (57.4, 105.0)	110.0 (80.6, 148.0)
**ALB(g/L)**	47 (45, 49)	46 (44, 48)
**SCr (μmol/L)**	78 (71, 85)	58 (53, 64)
**hypertension(%)**	34.3	19.7
**diabetes(%)**	11.4	4.1
**Smoking status (%)**		
** Smoker**	46.4	3.1
** Ex-smoker**	0.12	0.00
**Drinker (%)**	62.6	12.6
**Family history of diseases (%)**		
** CVD**	43.6	47.5
** Hypertension**	57.3	58.2
** Hyperlipidemia**	0.4	0.7
** Diabetes**	24.4	25.1

^a^ BMI, body mass index; TC, total cholesterol; TG, triglycerides; LDL, low density lipoprotein cholesterol; HDL, high-density lipoprotein-cholesterol; SBP, systolic blood pressure; DBP, diastolic blood pressure; FBS, fasting blood sugar; ALB, albumin; SCr, serum creatinine; IgM, immunoglobulin M; CVD, cardiovascular disease.

^b^ Median (interquartile range) (all such values).

**Table 4:** Results of Multivariate Modelling for change of IgM (n = 2,615) ^a^.

	Change of IgM (mg/dl)	
Males	Standard β coefficient (SEM) ^b^	*P* value ^b^
**Change of TG (mmol/L)**	0.882 (0.43)	0.04
**Age (y)**	0.478 (1.69)	0.78
**BMI (kg/m** ^**2**^ **)**	-0.234 (0.54)	0.67
**Hypertension**	-0.308 (0.72)	0.67
**Diabetes**	-1.089 (1.08)	0.31
**ALB (g/L)**	3.882 (4.69)	0.41
**SCr (μmol/L)**	-1.062 (2.37)	0.65
**Smoking status (%)**		
** Smoker**	-0.004 (0.70)	1.00
** Ex-smoker**	-2.513 (9.67)	0.80
**Drinker (%)**	0.37 (0.71)	0.60
**Family history of diseases (%)**		
** CVD**	-0.519 (0.69)	0.45
** Hypertension**	1.088 (0.70)	0.12
** Hyperlipidemia**	1.664 (5.60)	0.77
** Diabetes**	0.226 (0.79)	0.77
Females		
**Change of TG (mmol/L)**	0.821 (1.29)	0.53
**Age (y)**	-5.131 (2.95)	0.08
**BMI (kg/m** ^**2**^ **)**	0.422 (0.89)	0.64
**Hypertension**	-1.241 (1.70)	0.47
**Diabetes**	-2.948 (3.23)	0.36
**ALB(g/L)**	-15.772 (8.74)	0.07
**SCr (μmol/L)**	1.01 (4.29)	0.81
**Smoking status (%)**		
** Smoker**	1.835 (3.62)	0.61
**Drinker (%)**	-1.191 (1.89)	0.53
**Family history of diseases (%)**		
** CVD**	-1.571 (1.30)	0.23
** Hypertension**	-0.736 (1.30)	0.57
** Hyperlipidemia**	-14.565 (7.59)	0.06
** Diabetes**	-1.951 (1.46)	0.18

^a^ BMI, body mass index; TG, triglycerides; IgM, immunoglobulin M; ALB, albumin; SCr, serum creatinine; CVD, cardiovascular disease; SEM, standard error of the mean.

^b^ multiple linear regression analysis.

## Discussion

This cross-sectional and prospective cohort study demonstrates that serum concentration of IgM varies with serum TG levels in males, but not in females.

These results agree with previous studies showing that IgM levels are higher in females than males [22, 23]; based on this, we separated the study cohort by gender in order to analyze the relationship between IgM and TG levels. Higher IgM levels in females have been attributed to the stimulatory action of estrogens on B lymphocytes [24]. However, levels of sex hormones such as testosterone and estrogen were not measured in this study. Further research is needed to explore this issue. A number of studies have demonstrated that metabolic diseases including MS and T2D are more common in men than in women [25, 26]. Considering the higher levels of IgM, coupled with an apparent lower incidence of metabolic disease, it is reasonable to postulate that in females, many body tissues are more tolerant to the action of IgM. Other possible reasons that may account for lower rates of metabolic disease are differences in lifestyle and/or dietary patterns, both of which have been correlated with metabolic disorders [27, 28].

We adjusted for multiple potentially confounding factors in our analysis. This study suggested that numerous factors (BMI, waist circumference, drinking, smoking status, family history of some diseases) correlated positively with serum TG levels. Studies have shown that serum TG levels and inflammatory status are related to age and BMI, so we first adjusted for these two variables. Adjustment for age and BMI significantly affected the relationship between serum TG levels and IgM concentration: in males and females this adjustment reversed the results, leading us to conclude that age and BMI are major confounding factors. We subsequently adjusted for SCr, ALB, hypertension, diabetes, smoking and drinking status (influential factors on IgM levels [29]), and any family history of diseases including CVD, hypertension, hyperlipidemia, and diabetes (all of which are recognized as genetic factors for dyslipidemia [30]). After this adjustment, serum TG levels had more obvious correlation with serum IgM concentration in males.

Obesity-related chronic low-grade inflammation often gives rise to chronic diseases, such as MS, T2D and CVD. More recently, IgM has emerged as a possible mechanistic player underlying the pathophysiology of these diseases. IgM antibodies have low affinities and broad specificities to both foreign and self epitopes [12], and is thus is believed play a role in autoimmune disorders [31]. A cross-sectional study performed by Gonzalez-Quintela A et al., suggested that individuals with hypertriglyceridaemia exhibited higher IgM levels than individuals without it [22]. Another follow-up study showed a significant decrease in IgM levels in the early postoperative period accompanying slight weight loss following the Laparoscopic Adjustable Gastric Banding (LAGB) procedure [32]. These studies indicate that IgM and TG levels are increased in obese populations; however, few studies have investigated an association between serum TG levels and IgM concentration in an adult population. Our study is the first to show serum TG levels are positively and significantly related to serum concentration of IgM.

IgM is the first class of antibodies produced in response to antigen challenge, and is generated mainly by B-1 cells. One study using obese mice indicated that B cells in VAT are exposed to increased levels of fatty acids that have been shown to stimulate TLR-4 receptors on B-1 cells, a strong stimulus for increased IgM production [17]. Because TG is fatty acid carriers, the two compounds are closed related; thus, serum fatty acid may be the link between TG levels and IgM concentration. Moreover, it is known that oxidation-specific epitopes are dominant targets of natural IgM antibodies during chronic inflammation [18]. For example, IgM antibodies dominate the humoral response to epitopes of oxidized low-density lipoproteins cholesterol (Ox-LDL) in mice, and using mouse models of atherosclerosis [33]. Galle et al. [34] found that formation of Ox-LDL is promoted by oxidative stress. F2-isoprostanes formation is substantially increased in animal models of free-radical mediated injury [35] and is now used frequently as an accurate measure of oxidative stress in human studies. Moreover, F2-isoprostanes were significantly and positively correlated with TG levels in men but not in women [19]. Based on this finding, increased serum TG levels may promote oxidative stress in males, which, in turn, results in increased serum concentration of IgM. Further research is warranted to clarify whether or not other mechanisms are involved with TG-related increases in IgM levels.

Limitation of this study should be noted. First, because this was an observational study, we could not conclude causal relationships between TG and serum concentration of IgM. Therefore, an intervention trial should be undertaken to confirm the existence of a relationship between TG and IgM. Second, although we adjusted for hypertension and diabetes, the present study cannot completely eliminate the possible effects of other underlying diseases and medications used for these diseases on the results. Third, the sample size available for follow-up and the duration of the follow-up period were relatively small. Therefore, a larger-scale and long-term prospective study is necessary to verify the results reported here.

Our analysis provides exploratory evidence that IgM levels vary with increased serum TG levels in a gender-dependent manner. Numerous studies have indicated that higher serum TG levels were positively related to autoimmune diseases, such as autoimmune thyroiditis, systemic lupus erythematosus [36–38], and others have suggested that elevated serum IgM levels increased the risk of autoimmune diseases. We propose a hypothesis that TG elevation contributes to the biological processes regulating the immune system by increasing IgM, and that IgM inhibition is an attractive potential therapy to prevent metabolic disorders and obesity-related autoimmunity.

## Conclusion

This cross-sectional and prospective cohort study is the first to show that serum concentration of IgM varies with serum TG levels in males, but not in females. The findings suggest that IgM may be involved in the pathological process of lipid metabolism disorder. Further research is needed to explore the mechanism by which TG leads to increased IgM concentration.
